# Development of chromosomal markers based on next-generation sequencing: the B chromosome of the cichlid fish *Astatotilapia latifasciata* as a model

**DOI:** 10.1186/s12863-016-0427-9

**Published:** 2016-08-18

**Authors:** Bruno E. A. Fantinatti, Cesar Martins

**Affiliations:** Departamento de Morfologia, Instituto de Biociências, UNESP – Universidade Estadual Paulista, CEP 18618-689 Botucatu, SP Brazil

**Keywords:** Supernumerary chromosome, Molecular markers, Chromosome polymorphism, Evolution

## Abstract

**Background:**

B chromosomes (Bs) are additional chromosomal elements found in a wide range of eukaryotes including fungi, plants and animals. B chromosomes are still enigmatic despite being the subject of hundreds, even thousands of reports. As yet there is no comprehensive theory for the biological role of B chromsomes thus, new studies are needed. Next-generation sequencing (NGS) holds promise for investigating classical issues in chromosome biology. NGS uses a large-scale approach that is required for advancing classical cytogenetic studies. Based on 454 sequencing data of a microdissected B chromosome and Illumina whole-genome sequencing data generated for 0B, 1B and 2B animals, we developed PCR- and qPCR-based markers for the B chromosomes of the cichlid fish *Astatotilapia latifasciata* (that possess 0, 1 or 2 B chromosomes).

**Results:**

Specific PCR primers were designed to produce two amplified fragments for B-positive samples and the control fragment for B-negative samples. Thus, PCR markers detected the presence/absence of Bs but did not provide information about the number of Bs. However, quantitative PCR (qPCR) markers clearly discriminated between 1B and 2B samples. The high copy number of the marker identified in the B chromosomes was confirmed by chromosome mapping.

**Conclusions:**

The analysis of chromosome polymorphisms based on a NGS approach is a powerful strategy to obtain markers that detect the presence/absence of extra chromosomes or the gain or loss of genomic blocks. Further, qPCR can also provide information regarding the relative copy number of specific DNA fragments. These methods are useful to investigate various chromosome polymorphisms, including B and sex chromosomes, as well as chromosomal duplications and deletions. NGS data provide a detailed analysis of the composition of genomic regions that are thought to be present in B chromosomes.

## Background

B chromosomes (Bs) are one of the most astonishing features of chromosome biology. Approximately 15 % of eukaryotes contain B chromosomes, which are present in plants, animals and fungi [1]. Current knowledge on Bs indicates that these chromosomes arose multiple times by diverse mechanisms. Therefore Bs in different species may be completely diverse in terms of origin, evolution and genomic content. However, given that these chromosomes are mostly heterochromatic, Bs include a large amount of repetitive DNA [1]. B chromsomes are still enigmatic despite being the subject of hundreds, even thousands of reports. As yet there is no comprehensive theory for the biological role of B chromsomes thus, new studies are needed. Most of our knowledge on Bs derives from cytogenetical approaches. The preparation of chromosome spreads often depends on sacrificing animals. The development of alternative methods to assess genetic patterns that do not require euthanasia are desirable. The preparation of fish chromosomes generally involves stimulating cell division by yeast injection followed by kidney tissue extraction after sacrificing the animals. Complications can arise when using this method. For instance yeast injections can cause contamination, leading to immunological responses, and disrupting further functional analysis. Additionally, given that only kidney tissue is used for chromosome preparations, the presence of Bs in other tissues is not assessed and information may be lost. Although chromosome preparations can also be made from cell culture, this procedure is not well established for many species. Thus, the development of techniques to detect chromosome polymorphisms based on PCR without disturbing the cell profile and without necessitating euthanasia would be a distinct advantage.

Chromosome studies made rapid progress in the second half the 20th century after the introduction of molecular methods such as in situ hybridization of nucleic acids to chromosomes [2, 3]. As a consequence, molecular cytogenetics exhibit a wide range of applications in chromosomal biology. The development of fluorescent in situ hybridization [4], chromosome painting [5] and bacterial artificial chromosome (BAC) probes [6] were important advances in chromosome studies. Recent advances in genome sequencing offer the possibility to explore karyotypes and chromosomes based on the nucleotide sequences of whole chromosomes and genomes. The karyotype of species can be recnstructed based on *in silico* data of complete nucleotide sequences of genomes. These new approaches open the possibility of a powerful integration of genomics and cytogenetics to investigate chromosome biology. For instance, the ancestral synteny of nucleotide sequences across different taxa can be established based on sequence orthology among species. An application of this approach is electronic chromosome painting (E-painting), a type of “*in silico* cytogenetics”. This method provides a new tool for analyzing chromosomes and karyotypes [7], through the identification of conserved linkage groups even between very distantly related animal taxa [8, 9].

The development of next-generation sequencing (NGS) in the first decade of the 21st century changed the universe of genome sequencing. It has made low-cost and high-throughput sequencing available to ordinary laboratories and applicable to a wide range of species. NGS can be applied to a wide range of research questions. It allows rapid advances in many fields related to the biological sciences, such as sequencing genomes, comparative biology studies, public health, epidemiology, physiology, and gene expression. Here, we explore NGS methods to investigate a classical issues in cytogenetics, B chromosomes analysis. We developed conventional and quantitative polymerase chain reaction (PCR and qPCR, respectively) procedures based on NGS data to generate genetic markers to genotype the presence/absence and number of B chromosomes using the cichlid fish *Astatotilapia latifasciata* as a model. Our analysis shows that NGS data are useful in the development of DNA markers to investigate B chromosomes. Advances in the identification of Bs are helpful in clarifying several aspects of their enigmatic biology.

## Methods

### DNA samples and karyotyping

Specimens of the cichlid fish *Astatotilapia latifasciata* (native to lakes Kyoga and Nawampasa in Uganda, satellite lakes of Lake Victoria in Africa) were obtained from a stock established from the aquaculture trade and maintained in the fish facility of the Integrative Genomics Laboratory at Sao Paulo State University (Botucatu, Brazil). A total of 90 specimens (70 0B, 16 1B and 4 2B) belonging to the same aquarium population were analyzed. The experimental procedure was conducted according to the international guidelines of Sao Paulo State University and approved by the Institutional Animal Care and Use Committee (IACUC) (Protocol no. 34/08 - CEEA/IBB/UNESP). The animals were euthanized through immersion in a water bath with 250 mg/l benzocaine for 10 min. The animals were karyotyped by classical cytogenetic procedures using Giemsa stain to identify the 0B, 1B and 2B karyotypes as previously described [10, 11]. DNA samples of 0B, 1B and 2B animals were extracted from fin clips and stored at −80 °C for the next steps of analysis.

### Genome data

Previous genomic sequencing of the microdissected B chromosome using the 454 platform resulted in a total of 125,601 reads comprising 48,637,895 base pairs (bp) [12]. The assembly resulted in 3836 contigs with an average size of 372 base pairs. Generated contigs were compared with the National Center for Biotechnology Information (NCBI) nucleotide collection [13]. The contigs with hits against fish DNA sequences were retrieved for further analysis.

Datasets of Illumina sequencing generated from 0B (401,017,570 reads), 1B (296,161,988) and 2B (306,823,512 reads) male samples were aligned to the *Metriaclima zebra* cichlid reference genome using Bowtie2 software [14]. The 0B and 2B datasets were obtained from [12], and the 1B genome was sequenced de novo. The coverage ratio of read alignments the 0B, 1B and 2B samples was analyzed to identify increased coverage regions exclusive to B genomes according to [12]. All sequences and alignments are available in the genome browser of the SaciBASE database [15].

### B chromosome markers development: B presence/absence

**Fig. 1:**
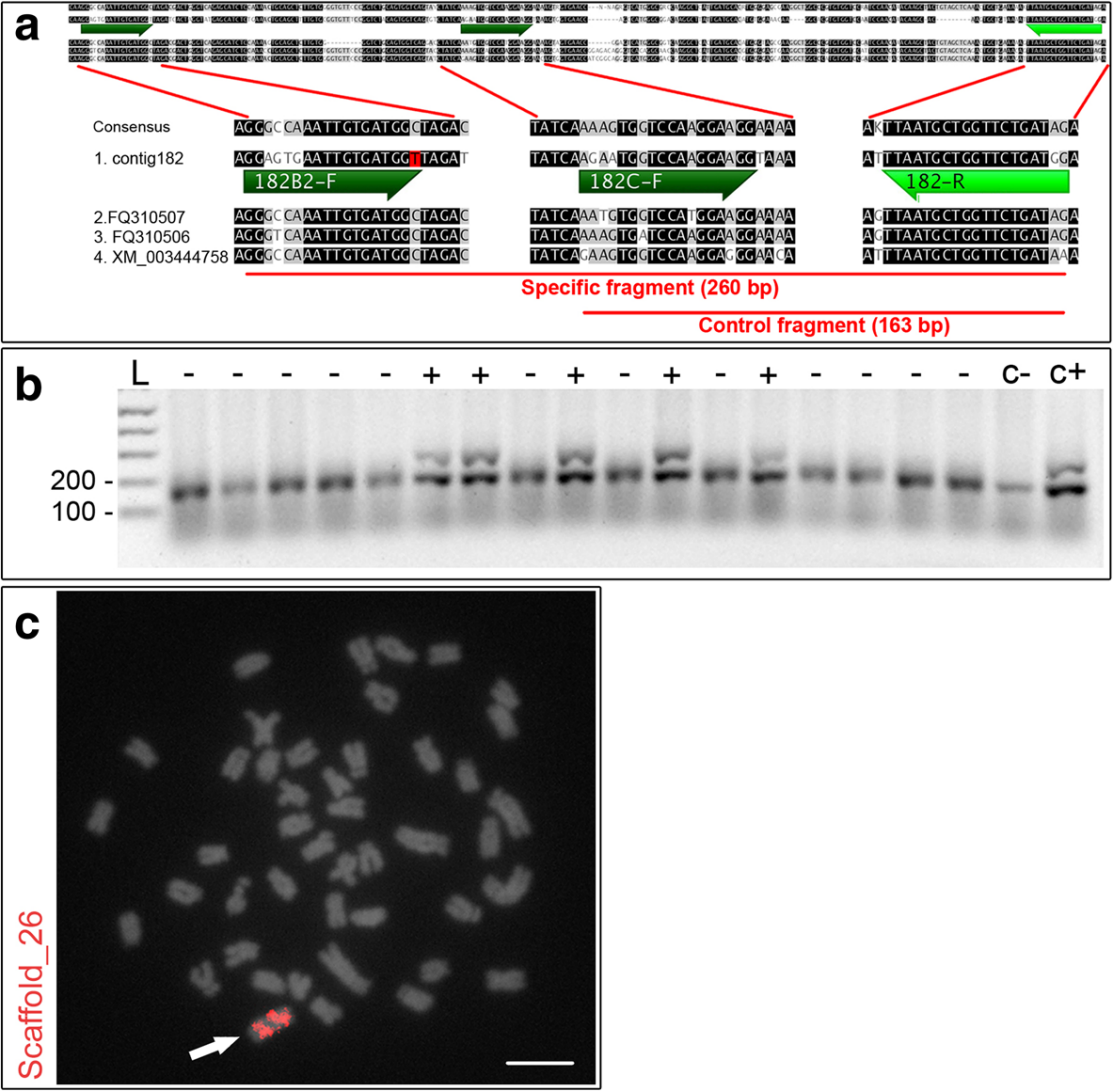
Design and results for the qualitative PCR markers. **a** Scheme for the primer design with emphasis on the B-specific and control fragments of scaffold_26. Three genomic sequences of different fishes: *Dicentrarchus labrax*, *Dicentrarchus labrax* and *Oreochromis niloticus* (FQ310507, FQ310506, and XM_003444758, respectively) were used to establish a consensus for comparison with the 454 sequencing data of the microdissected B chromosome (contig_182). **b** A 1 % agarose gel showing PCR products from B-positive (+) and B-negative DNA (−) samples. Note that the B- samples present only one DNA fragment (control fragment), whereas the B+ samples present two fragments (the control fragment and a B-specific fragment). **c** FISH using the PCR marker region sequence based on scaffold_26 as a probe. An *arrow* indicates the B chromosome, and the scale bar indicates 5 μm

For the development of conventional PCR markers to detect B chromosome presence/absence, contigs generated from 454 sequencing were aligned against the cichlid genome data available at NCBI. A total of 31 different oligos were designed over nine different contigs that aligned to the cichlid genomes (Table 1). Conserved regions between B genomic data and cichlid genomes were retrieved as a control for the reaction. Primers were designed over the conserved regions to amplify a control fragment regardless of B chromosome presence (Fig. 1a). Subsequently, a third oligo was designed with its 3′ end exactly over the nucleotide variation that is characteristic of the B genome to amplify a DNA fragment only in the presence of B chromosome DNA (Fig. 1a). The oligos were designed to work in a multiplex reaction to amplify only one fragment in B-negative animals (the control fragment) and two fragments in B-positive samples (the B-specific fragment plus the control fragment).

**Table 1:** Primers for qualitative PCR markers

Primer ID	Primer sequence
49B1-F	5′ GAGCTTCACACTTGCAGAGGTAAGTCATTTTTGCAGAGAC 3′
49B2-F	5′ GCTTCACACTTGCAGAGGTAAGTCATTTTT 3′
49C + −F	5′ GTTTACAGTCTGATGATGGGACATCATGCTCTGC 3′
49-R	5′ TGTCCAGAGTATAATCGCAGCCTTTGCGGT 3′
80B-F	5′ GAGGCATTACATCGGTCTTTCCATCA 3′
80C + −F	5′ GGTGAGCAGCAGGATTTTGAATTGAATGCG 3′
80-R	5′ CCTGATTGAGTGCTTCTCACAC 3′
182B1-F	5′ GGGTGTGTTTGGTTGTGGTTTGACAAGGAGTG 3′
182B2-F	5′ GGAGTGAATTGTGATGGT 3′
182B3-F	5′ GGTTTGACAAGGAGTGAATTGTGATGGTTAGATC 3′
182B4-F	5′ GAGTGAATTGTGATGGTTAGATCACTAGGTAT 3′
182C + −F	5′ AGAATGGTCCAAGGAAGG 3′
182-R	5′ CCATCAGAACCAGCATTAA 3′
207B-F	5′ GAGACACTTCTTGGAGAAAATGAAATGCCCAC 3′
207C + −F	5′ ACCAGGCCAGGAGACGACTGAAGAACT 3′
207-R	5′ GACCTGCAGAAATGTGAACATGGTTGCAGTTTACAA 3′
323B-F	5′ GGGGGTGTTTTGCTTTTGGTTTTCCTACATTAGTTA 3′
323C + −F	5′ GTATAAGCCATCTCTGTCATCTAAGGTACA 3′
323-R	5′ GACACAGTACAGCTGACACAGACGAAGCAACAG 3′
764B-F	5′ CCTGAGATGGTCCGATTGGGCTGGTAA 3′
764C + −F	5′ GGTGAAGCATCAAAGAGCTCTCTGAGTCT 3′
764-R	5′ GGAGACAAGGAGATGCGTGTTGGTGAAGTCCTAA 3′
1100B-F	5′ GGGTGTGTGGAGATGTACATCAGCACACATGTT 3′
1100C + −F	5′ CACTGAGACGGCATTGGCATGAGAAA 3′
1100-R	5′ AGCATGGTGGCAGAGGTCTTTA 3′
1987B-F	5′ CCCTCCTGTTATTCATTCCCTA 3′
1987C + −F	5′ TACTTTGCTGTGTGTTTTGCCTGTC 3′
1987-R	5′ AAGTGTGGCTGTGTGCAGGCAGGAAT 3′
2519B-F	5′ GCAGGATTCAGGAGTGAAGCATCTGTGTGA 3′
2519C + −F	5′ CACTAAACTGCAGACATCAGGCTG 3′
2519-R	5′ CATTGTTCTGCTGCAGTCAATGGAC 3′

The numbers 49, 80, 182, 207, 323, 1100 and 1987 are references to the contigs obtained with 454 sequencing data

The letters B and C indicate the specificity of the primer as a B-specific primer or positive control (B or C+), respectively, followed by the reverse/forward annotation (F or R)

The optimal annealing temperature for 31 oligos was assessed using a gradient thermocycler ranging from 45 to 65 °C. The reactions were performed as follows: 1 U Platinum Taq DNA Polymerase, 1.5 mM MgCl_2_, 1 × RXN Buffer, 0.32 mM dNTP, 0.2 μM of each primer (totaling 0.6 μM) and ultrapure water up to 25 μl. Cycling was performed as follows: 5 min at 95 °C, 34 × (1 min at 95 °C, 30 s at 50 °C, and 45 s at 72 °C), and 5 min at 72 °C. The material was then analyzed in a 1 % agarose gel.

### B chromosome marker development: number of Bs

Illumina reads from the 0B, 1B and 2B datasets were separately aligned to the cichlid *Metriaclima zebra* reference genome (available at [13]) using Bowtie2 software [14]. The alignment results were subjected to coverage ratio analysis among 0B, 1B and 2B datasets according to [12]. Given that B chromosomes present a high number of repetitive DNA sequences, the genomic regions of the B chromosome presenting an increased copy number would present a higher coverage rate compared with regular A chromosomes. Thus, a total of six genomic regions with high coverage in the 1B and 2B genomes compared with the 0B genome were selected for qPCR analysis (Table 2). qPCR primers were designed over those regions (Table 3). qPCR experiments were performed using known DNA samples previously genotyped by a cytogenetic approach, i.e., three of each 0B, 1B and 2B samples. Such samples were used as controls in all qPCR genotyping procedures.

**Table 2:** Selected genomic regions for qPCR primer design. The scaffolds can be accessed in the SaciBase database

Scaffold	Start position	End position	Size (bp)
Scaffold_3	9112721	9126380	13,659
Scaffold_13	5682225	5684956	2731
Scaffold_19	960417	963148	2731
Scaffold_26	1707513	1710244	2731
Scaffold_31	5673821	5680650	6829
Scaffold_324	77097	77779	682

**Table 3:** qPCR primer set designed over six high-coverage genomic regions

Primer ID	Primer sequence
Scaffold_3-F	5′ GCCACCATGTTCAGATTATTGGAGAGTA 3′
Scaffold_3-R	5′ AATGCCTGACTTATCCATGCCAGGTG 3′
Scaffold_13-F	5′ CGTTTTGTACGTCTGCTGGA 3′
Scaffold_13-R	5′ ACCGGTACCTGTGGTCTAGT 3′
Scaffold_19-F	5′ TGGAGCATGAGTCGAAAAGCA 3′
Scaffold_19-R	5′ TCGCAGAACAGTGTGAACCA 3′
Scaffold_26-F	5′ AGACGGGTCGGGATCTTACA 3′
Scaffold_26-R	5′ TGTTTGAGCATCCCCCAGAC 3′
Scaffold_31-F	5′ CCAAGGCTCAGGAAATAGGGG 3′
Scaffold_31-R	5′ ACCACTGCTTCTCAAAGAGGG 3′
Scaffold_324-F	5′ CAGGTCCCTCTGCGTAACTG 3′
Scaffold_324-R	5′ GACGCCCCAGTCATCATTCA 3′

The qPCR experiments were conducted as follows. DNA samples were diluted to 30 ng/μl, and the expected concentration was confirmed using a Nanovue Spectrophotometer (GE). The reaction mix was prepared using 1× GoTaq qPCR Master Mix (Promega) with 0.16 μM of each primer, 20 μl of DNA (30 ng/μl) and ultrapure water up to 75 μl. The reactions were run in triplicate (three of each 0B, 1B and 2B known samples). The hypoxanthine phosphoribosyltransferase gene (HPRT), which is widely used in qPCR experiments, was used as a control gene for the analysis of the obtained data based on gene dose ratio analysis (GDR) using 2^-∆Ct^ [16].

After the first round of PCR and qPCR tests using three known samples for each genotype, unknown samples were analyzed using only one sample for each control, including the qualitative PCR and the quantitative qPCR genotyping. In the last round of tests, DNA samples of different tissues (liver, brain, muscle, eye and heart) were collected and analyzed to test for the presence of B mosaics among the tissue sampled.

### Marker sequences characterization

For a better characterization of marker sequences, two rounds of BLAST [13] searches were performed. The first search considered the amplicon sequences for both markers (260 bp for the qualitative marker and 89 bp for the quantitative marker). The second search was based on the amplicon sequences plus 1 kb up and downstream for better knowledge regarding the flanking regions of the markers. Searches based on RepeatMasker [17] were also performed for both amplicons including the 1-kb flanking regions.

### Fluorescence in situ hybridization

DNA fragments of genomic regions of the B markers were mapped onto chromosomal complement-containing B chromosomes of *A. latifasciata* by fluorescence in situ hybridization (FISH). FISH was performed using the protocol described by [4] with modifications by [18]. After hybridization, the metaphase stage of *A. latifasciata* was analyzed using an epifluorescence Olympus BX61 microscope (Olympus, Tokyo, Japan), and the images were captured using an Olympus DP73 system.

## Results

### Marker development

Of all the sets of PCR primers designed for the 454 data (Table 1), the best amplifications were obtained using primers designed in the contig_182 (Fig. 1a) that corresponds to the scaffold_26 of the *M. zebra* reference genome. Other primer sets did not work properly, resulting in amplifications for only the control fragment, the B positive fragment or neither fragment.

The application of contig_182 primers resulted in fragments of the expected size for both the control and specific fragments, i.e., 163 bp for the control fragment, which appears in all samples, and 260 bp for the B-specific fragment, which appears only in B-positive (B+) samples (Fig. 1b). Considering the length divergence between the control and the B-specific fragment, B+ and B negative (B-) samples were clearly distinguished. The FISH experiment using the B-specific fragment of contig_182 as a probe reveals significant accumulation of such DNA throughout the entire length of the B chromosome, but no signal was observed in the A complement (Fig. 1c).

**Fig. 2:**
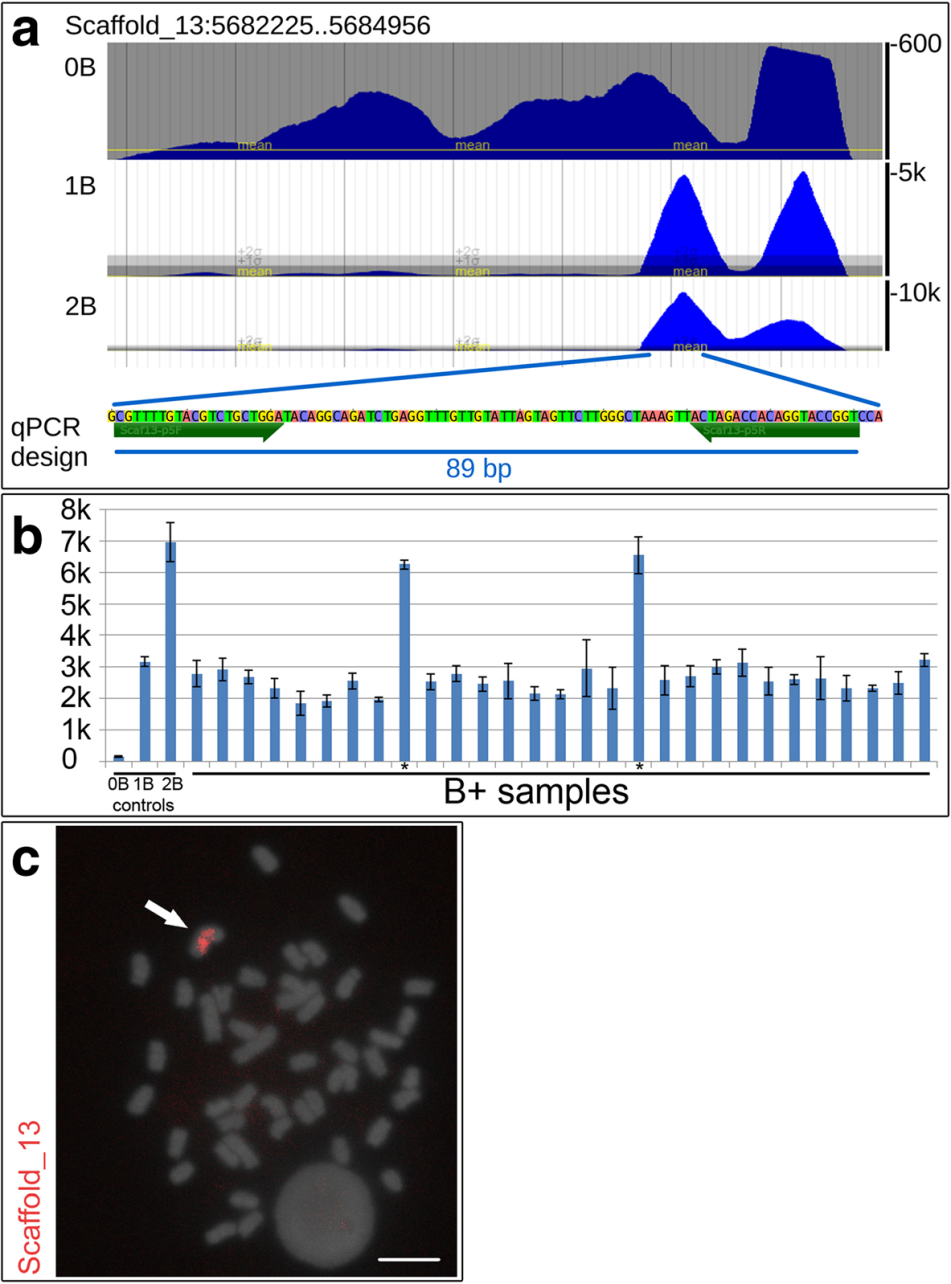
Development of and results observed for the qPCR marker analysis based on scaffold_13. **a** Scheme for primer design over a specific genomic region on scaffold_13 of 0B, 1B and 2B samples. Note the higher coverage for 1B (5,000× coverage) and 2B (10,000× coverage) samples compared with the 0B sample. **b** Graphic plot depicting the relative number of copies detected among B+ samples (1B and 2B) and control samples (0B). Note that 2B samples (*asterisks*) present approximately two-fold as many copies compared with the 1B samples. The standard deviation is presented for each sample. **c** FISH using the qPCR marker region sequence as a probe. An *arrow* indicates the B chromosome, and the scale bar is 5 μm

All B+ animals previously genotyped using the qualitative method based on 454 genome data were then used to proceed to the next step of genotyping, which was a quantitative PCR method based on the Illumina whole-genome datasets for 0B and 2B samples. Considering the genomic coverage ratios investigated by [12], six regions (scaffolds 3, 13 19, 26, 31 and 324) with higher coverage ratios in 2B genomes were selected for analysis (Table 2a). The coverage ratio was also confirmed in the 1B genome. The region corresponding to scaffold_13 of *M. zebra* (Fig. 2a) presented the best amplification rates, with low standard deviation values between the samples. All the others regions (scaffold_3, 19, 26, 31 and 324) presented amplification, but the standard deviations for the qPCR signals within these regions were very high. Thus, scaffold_13 was selected for sample analysis. The qPCR reaction results were efficient in the relative differentiation between 1B and 2B samples based on nine known control samples (three of each 0B, 1B and 2B samples) for each test. As expected, the relative copy number of the region selected for the tests was increased by approximately two-fold in 2B samples compared with 1B samples (Fig. 2b). The results indicate that the marker was able to differentiate between 1B and 2B genomes using this region as a reference. The FISH experiments using the quantitative qPCR marker fragment as a probe showed a very clear accumulation of the elements within the B chromosome, and no signals were observed in any of the A complement chromosomes (Fig. 2c).

### Genomic content of marker DNA fragments

For the qualitative marker, the amplicon region was used in a BLAST search, and the results reveal fragmented short-hits with similarities to the (i) vasa gene (284/320, 89 %. E-value: 7e-101), (ii) neuroligin-3-like gene (165/214, 77 %. E-value: 7e-21), (iii) zinc finger protein 845-like gene (of *Oreochromis niloticus*) (234/304, 77 %. *E*-value: 3e-35) and (iv) manganese superoxide dismutase gene (of *Hemibarbus mylodon*) (114/139, 82 %. E-value: 2e-21). A BLAST search using the quantitative marker amplicon fragment alone as a query did not return any relevant results. Thus, a fragment composed of the amplicon plus 1 kb upstream was analyzed. It was not possible to analyze 1 kb downstream of this region because that genomic region is not assembled in the reference genome. The BLAST search returned similarities to the growth hormone 2 gene of *Salmo salar* (95/116, 82 %. E-value: 4e-16) and the endogenous virus ERV-Pb1 envelope polyprotein gene of *Macaca nemestrina* (98/122, 80 %. E-value: 55–15).

A search using the RepeatMasker algorithm did not reveal any relevant results when using the amplicon sequences alone as a query. Searches using the amplicons plus 1-kb flanking regions as queries revealed the presence of known repeat segments for short interspersed nuclear elements (SINE) and long interspersed nuclear elements (LINE) for the qualitative marker and long terminal repeats (LTR) for the quantitative marker.

## Discussion

Recent discoveries have described a large number of genes encoded by the B chromosome and their influence in the transcriptional profile of the entire cell [19–21]. Current studies have used NGS methods to uncover B chromosome biology in the rye plant *Secale cereale* [22], the cichlid fish *Astatotilapia latifasciata* [12] and the fungal wheat pathogen *Mycosphaerella graminicola* [23]. Recent advances in NGS technologies have directed classical cytogenetics to the use of massive data analysis, allowing investigations of structural and functional issues at a level not previously possible. Thus, NGS analysis based on transcriptomes and microRNAomes are effective approaches to identify B chromosome functions because such techniques can directly indicate alterations that are not possible to detect through morphological traits. Thus, genotyping procedures for chromosome polymorphisms, based on simple analysis of DNA samples easily obtained from blood or any other tissue without sacrificing or harming the animal, are useful for gene expression studies.

Molecular cytogenetics has clarified some evolutionary issues by identifying sequences shared between A and B chromosome complements. Most species have B chromosomes that originated from its own A chromosome set. Classical and molecular cytogenetics approaches applied to B chromosomes have reached a limit in terms of data that can be obtained and the hypotheses that can be tested. It is now necessary to employ new methods and new technologies in order to advance our knowledge of B chromosomes. NGS technologies and the quality of the data obtained are consistently improving. There are various next-generation platforms, and each one presents specific advantages, varying from long read fragments to very high coverage capacity. In addition to the classical application of NGS to recover whole genomes, the paired-end approach can locate chromosomal breakpoints [24]. NGS aids in the investigation of chromosomal rearrangements involved in both speciation [25] and diseases [24]. Mapping chromosomal breakpoints is an essential dataset to clarify the evolutionary relationships between different taxa and quantify biodiversity - issues that have been classically addressed through chromosome painting [26].

As observed in our data, the qualitative and quantitative markers fragments in the B chromosomes are present in a high number of copies. This fact makes it possible to study these features in B chromosomes with FISH. However, in the A complement these same fragments are present as small, low copy number below the resolution of FISH. This limitation is overcome with the methods outlined here. The markers developed here were very efficient in revealing information related to B chromosome presence and quantity. The qPCR marker showed twice the relative amount of the fragment in 2B samples compared with 1B samples, indicating that such markers can be easily used for B chromosome quantification based on qPCR procedures. Such qualitative and quantitative genotyping procedures can contribute to the separation of animals into groups according to their genotypes, i.e., the number of Bs, for further analysis concerning B chromosome segregation through directed crossing. These procedures also provide a more accurate content analysis of B chromosome sequences through massive sequencing efforts.

These methods in addition to providing markers for B chromosome analysis are able to detect a huge number of sequences and compare such data against known databases. Issues regarding the content of such chromosomes can be better investigated. Cytogenetic procedures have been classically used to analyze B chromosomes [27]. Large-scale analysis of nucleotide sequences provides more detailed information about B chromosome composition [12, 22]. Although the selected marker regions do not present any functional annotation, the fact that these regions are surrounded by fragments of transposable elements corroborates the hypothesis that the accumulation of repeated sequences occurs during the formation and evolution of B chromosomes [11, 12]. Transposable elements are one of the most important keys for genome diversification and can influence the trajectory of evolution [28]. The expansion of repetitive copies in B chromosomes might reflect the lack of or low selective pressure present against the duplicated segments of B chromosomes. Furthermore, the presence of LTR as well as non-LTR SINE and LINE repeats in the flanking regions of the markers also corroborates the theory that transposable elements have involved in B chromosome evolution.

The presence of gene fragments in the marker regions (vasa, neuroligin-3-like, zinc finger protein 845-like, manganese superoxide dismutase, growth hormone 2 and endogenous virus ERV-Pb1 envelope polyprotein) is not surprising given that thousands of gene fragments, which were previously described for the B chromosome of *A. latifasciata* [12]. We cannot exclude the possibility that B chromosome duplication and expanded DNA copies, including gene fragments, have a functional role. We should note that noncoding RNAs potentially originated from genome duplications [29] and the deletion and insertion of segments [30]. These features might have an effect on genome functions. We might consider these duplicated regions as candidates to generate non-coding RNAs. Such noncoding segments can have a role in altering genome activity by modifying gene expression, as previously observed for the Bnc-RNA that originated from the B chromosome of *A. latifasciata* [31].

## Conclusions

Advances in genomics technologies and their integration with cytogenetics tools provide opportunities to investigate chromosome biology with rapid, larger-scale methods. B chromosomes are present in approximately 10–15 % of karyotyped eukaryotes. Their detection prior to whole-genome sequencing is auspicable to avoid disturbances in the assembly procedures. Further, the NGS data can provide information on the relative copy numbers of specific DNA segments that is complementary to FISH mapping to provide a more precise view of the genome.

Sequences obtained using NGS technologies can also aid in a more in depth analysis of the composition of genomic regions that are thought to be present in B chromosomes. The genome segments that have contributed to B chromosome formation and evolution can be determined by an alignment analysis of the generated sets of reads against a reference genome. Such an approach can be applied to other species and might result in better characterization of B chromosome content. The development of simple PCR-based genotyping procedures is also a welcome feature for future functional studies in *A. latifasciata*, as this method avoids disturbances in the transcriptional profile of cells.

## Abbreviations

BAC: Bacterial artificial chromosome
FISH: Fluorescence in situ hybridization
NGS: Next generation sequencing
qPCR: Quantitative PCR
